# Supplementary material for: Dual Blockade of Misfolded Alpha-Sarcoglycan Degradation by Bortezomib and Givinostat Combination
Source: Front Pharmacol. 2022 Apr 27;13:856804. doi: 10.3389/fphar.2022.856804 (PMC9093689; doi:10.3389/fphar.2022.856804)

Uncropped Western Blots

Figure 2D (alpha-sarcoglycan)

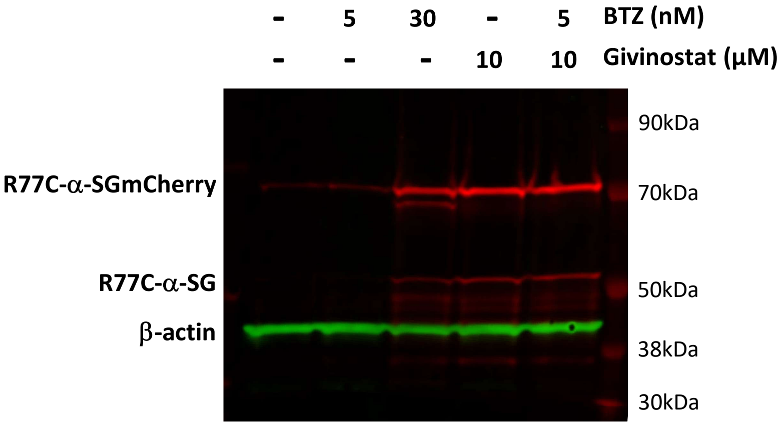

Figure 4E (CFTR)

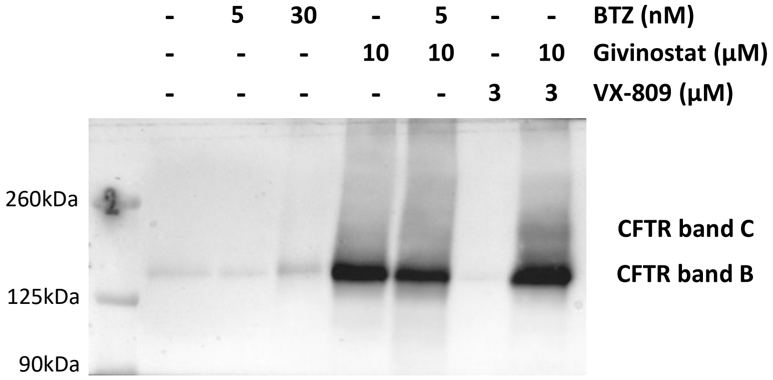

Figure 4E (Lamin B1)

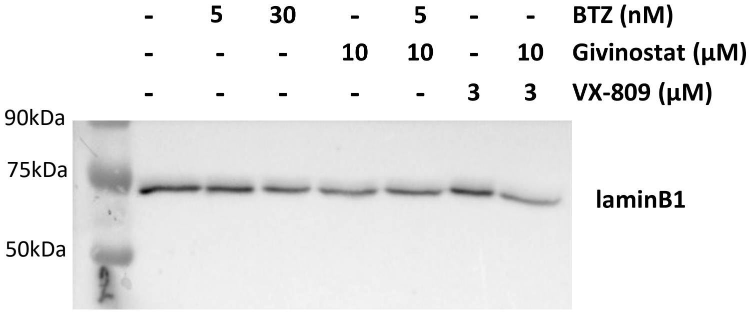

Uncropped Western Blots

Figure 5A (acetylated lysine)

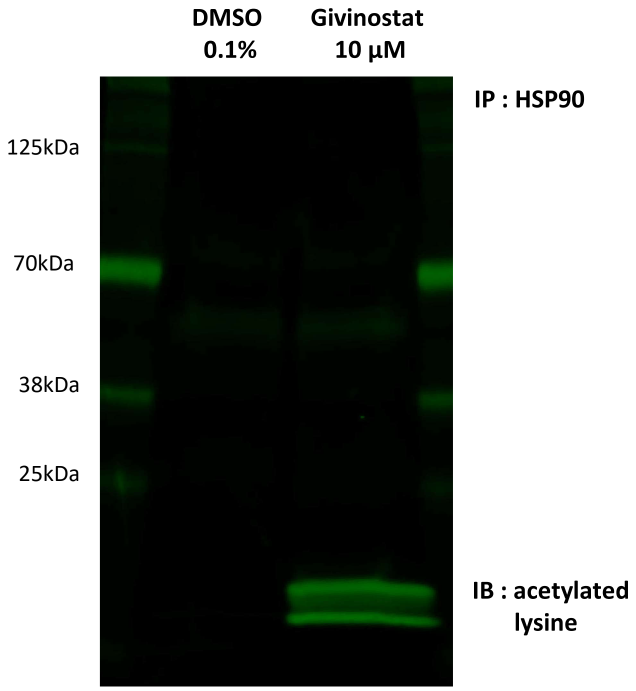

Figure 5A (HSP90)

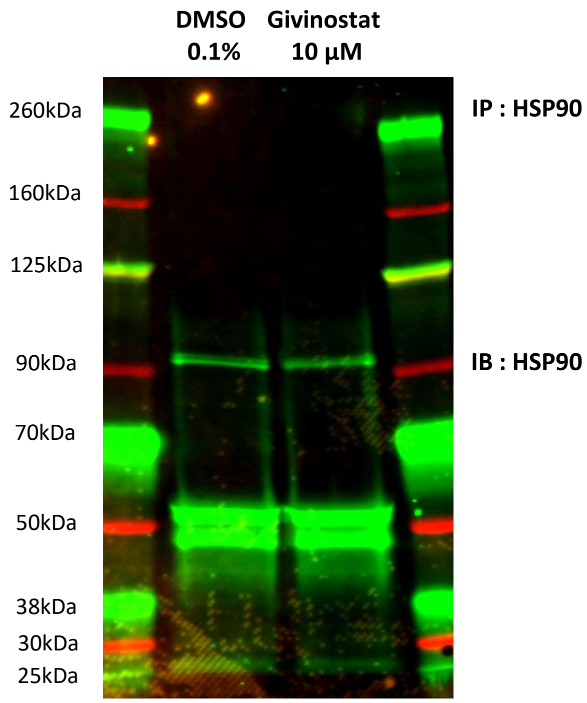

Figure 5B (alpha-sarcoglycan)

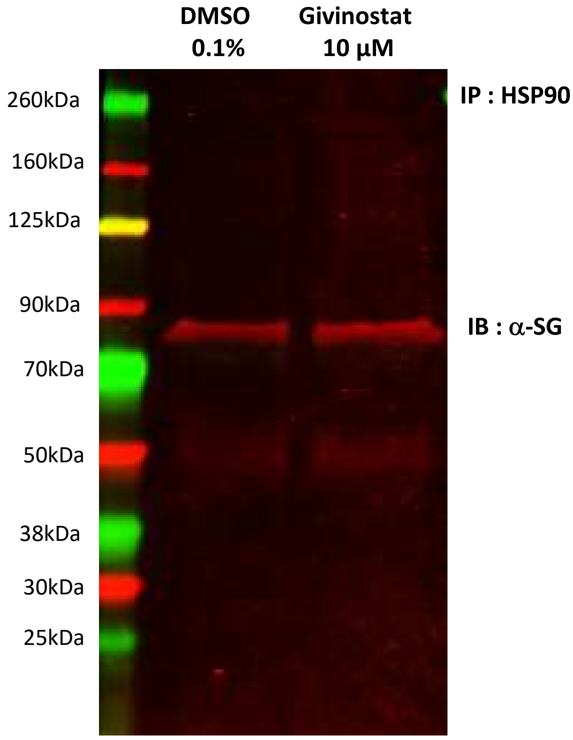

Figure 5B (HSP90)

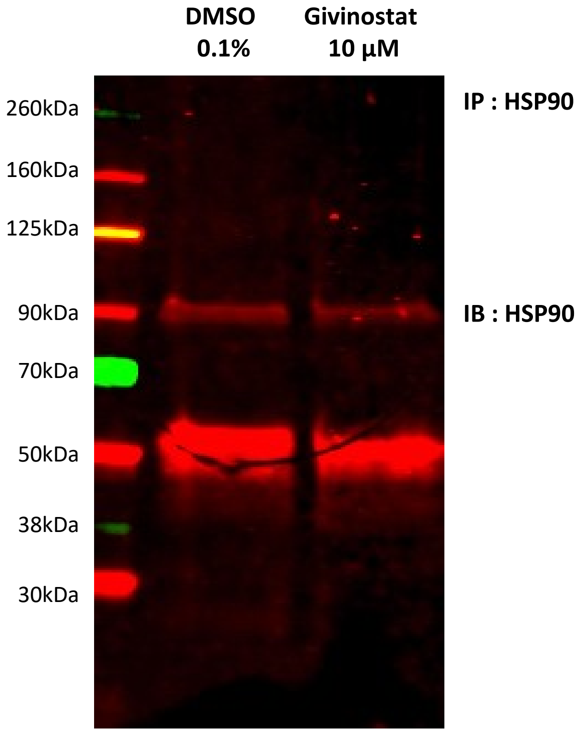

Uncropped Western Blots

Figure 5C

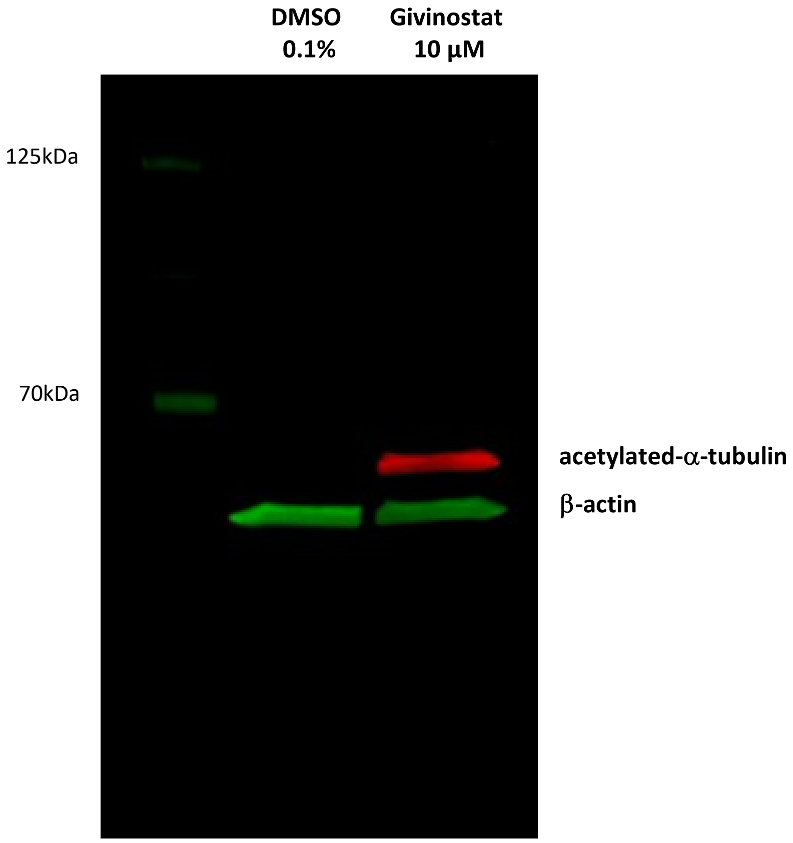

Figure 6A (ubiquitinated proteins)

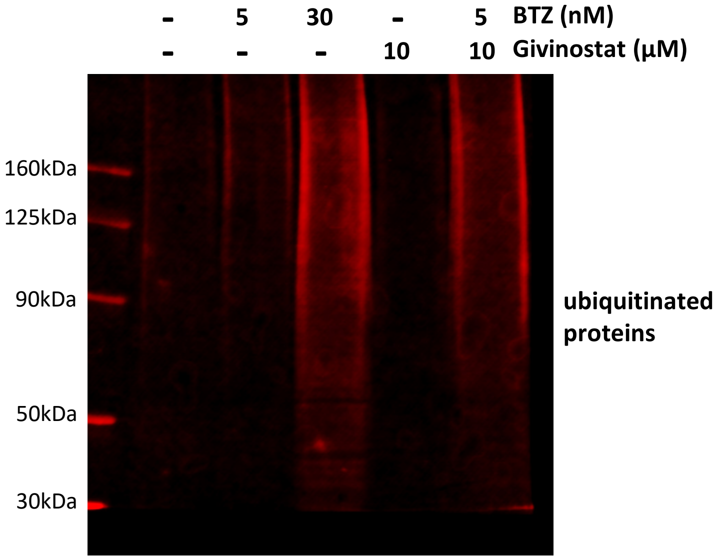

Figure 6A (LC3B)

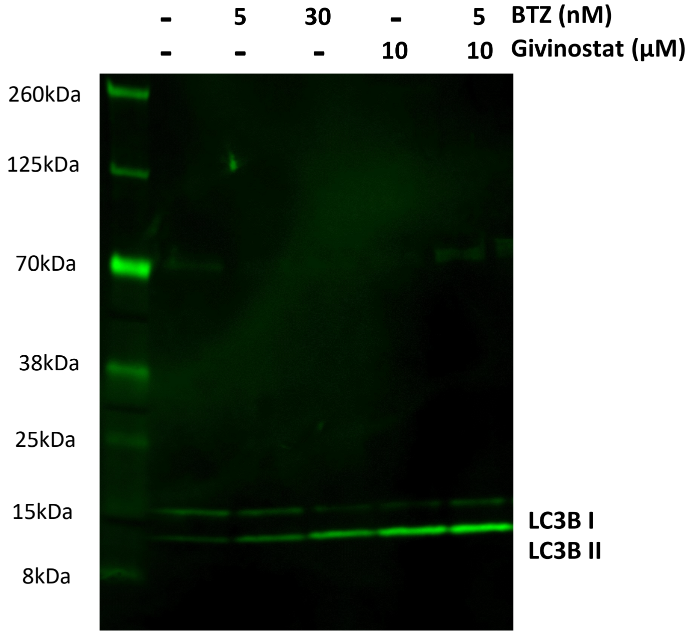

Uncropped Western Blots

Figure 6A (P62)

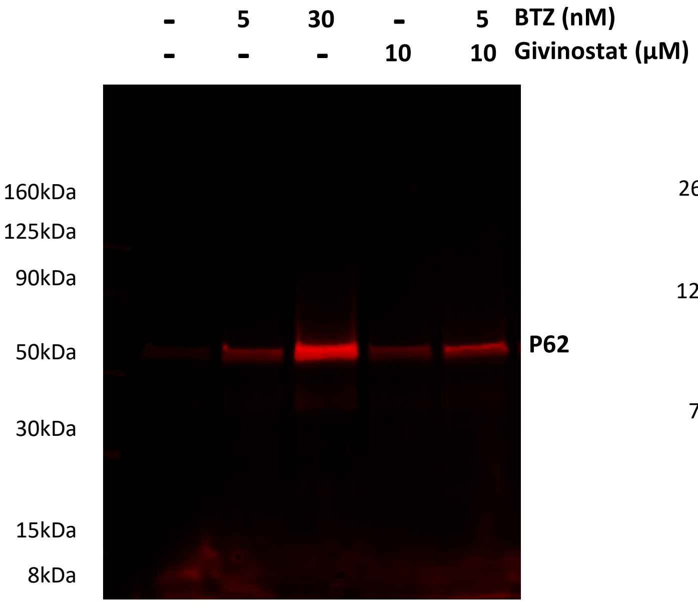

Figure 6A (β-actin)

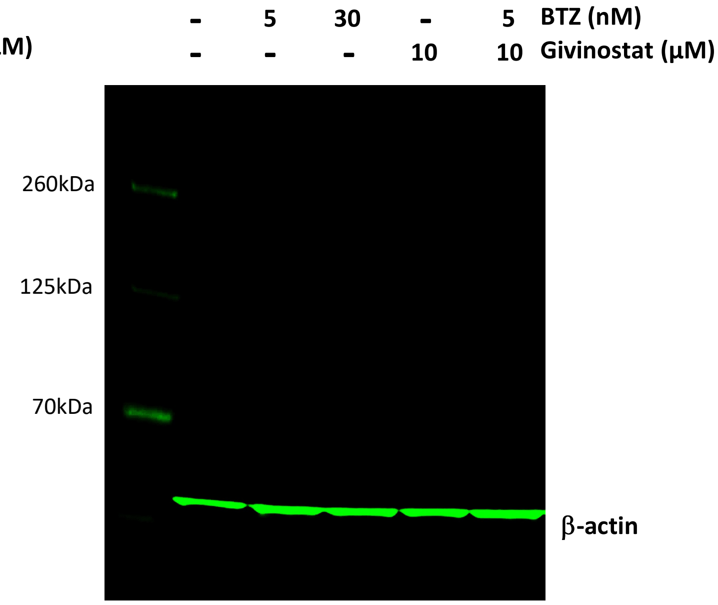

Figure 7C (V0a1)

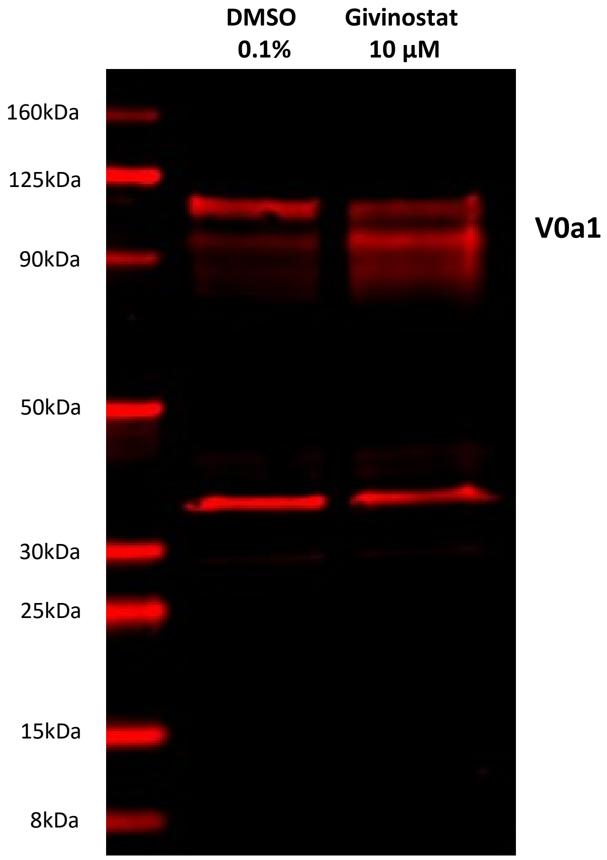

Figure 7C (β-actin)

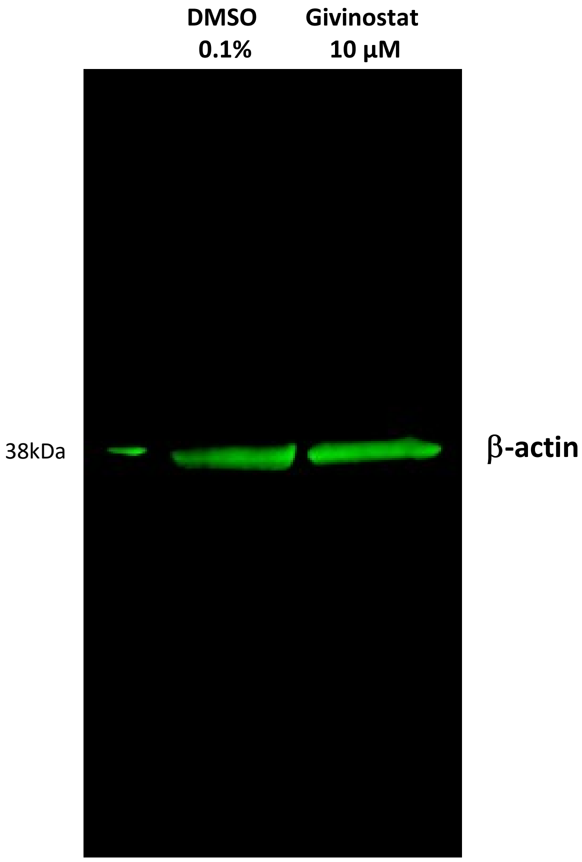

Uncropped Western Blots

Figure S2D (R77C-α-SGmCh)

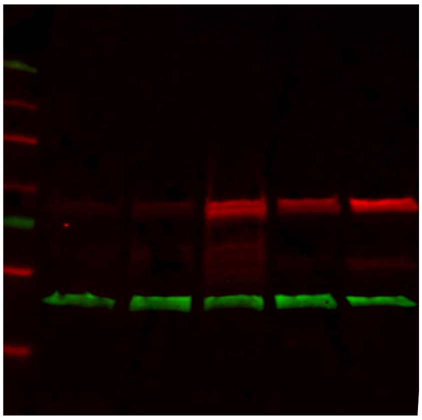

Figure S4D (R77C-α-SGmCh)

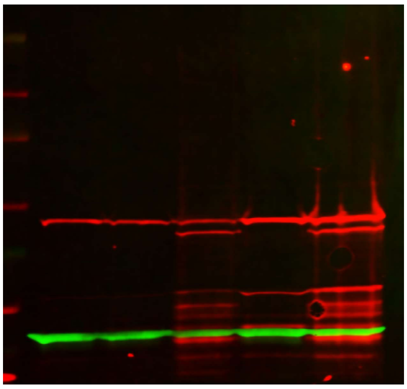

Figure S4D (acetylated-α-tubulin)

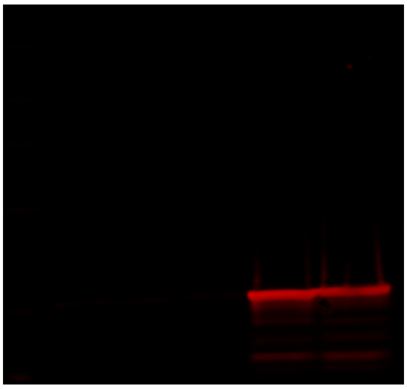

Supplement: Supplementary file 1 [file DataSheet2.pdf]
