# Supplementary material for: Dual Blockade of Misfolded Alpha-Sarcoglycan Degradation by Bortezomib and Givinostat Combination
Source: Front Pharmacol. 2022 Apr 27;13:856804. doi: 10.3389/fphar.2022.856804 (PMC9093689; doi:10.3389/fphar.2022.856804)
Supplement: Supplementary file 2 [file DataSheet1.pdf]

Supplementary Figure S1

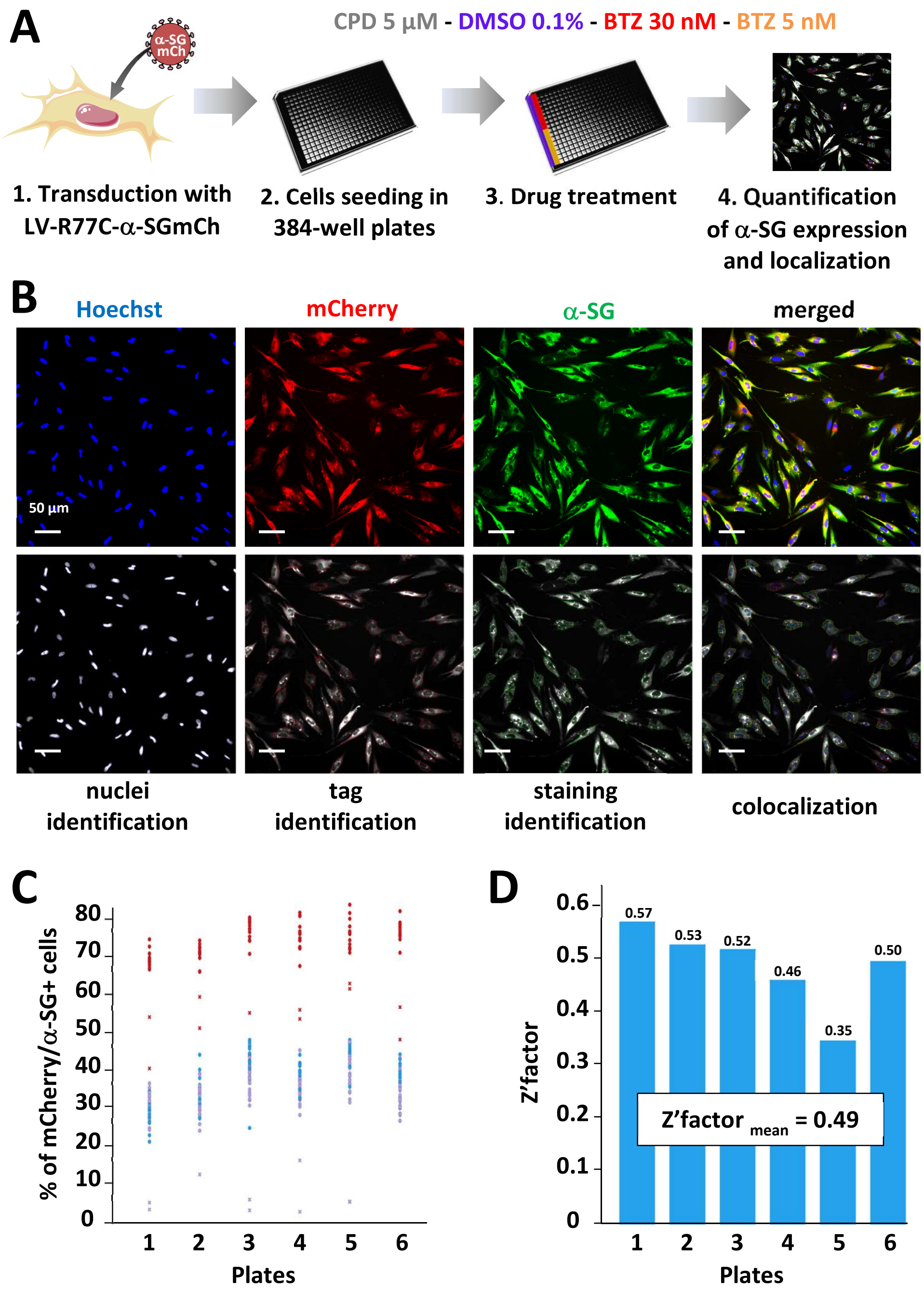

Supplementary Figure S1. Primary screen cell-based assay for R77C-α-SGmCh membrane rescue (related to Figure 1). (A) Workflow for the combinatorial high-content screening of R77C-α-SGmCh membrane rescue expression in 384 well plates. (B) mCherry fluorescent signal, membrane α-SG and Hoechst staining in fibroblasts overexpressing R77C-α-SGmCh and treated for 24 hours of treatment with BTZ 30 nM (top panels), and the corresponding masks images (bottom panels) allowing automated quantification of mCherry and membrane α-SG positive cells by colocalization. Scale bar = 50µm. (C) High-throughput screening validation for the R77C-α-SGmCh membrane rescue with the negative control, 0.1% DMSO, and the positive control, 30 nM BTZ, in each of the 384-well plates of the screening. (D) Determination of the Z′ factor in each of the 384-well plates of the screening. CPD = tested compounds; BTZ = bortezomib.

Supplementary Figure S2

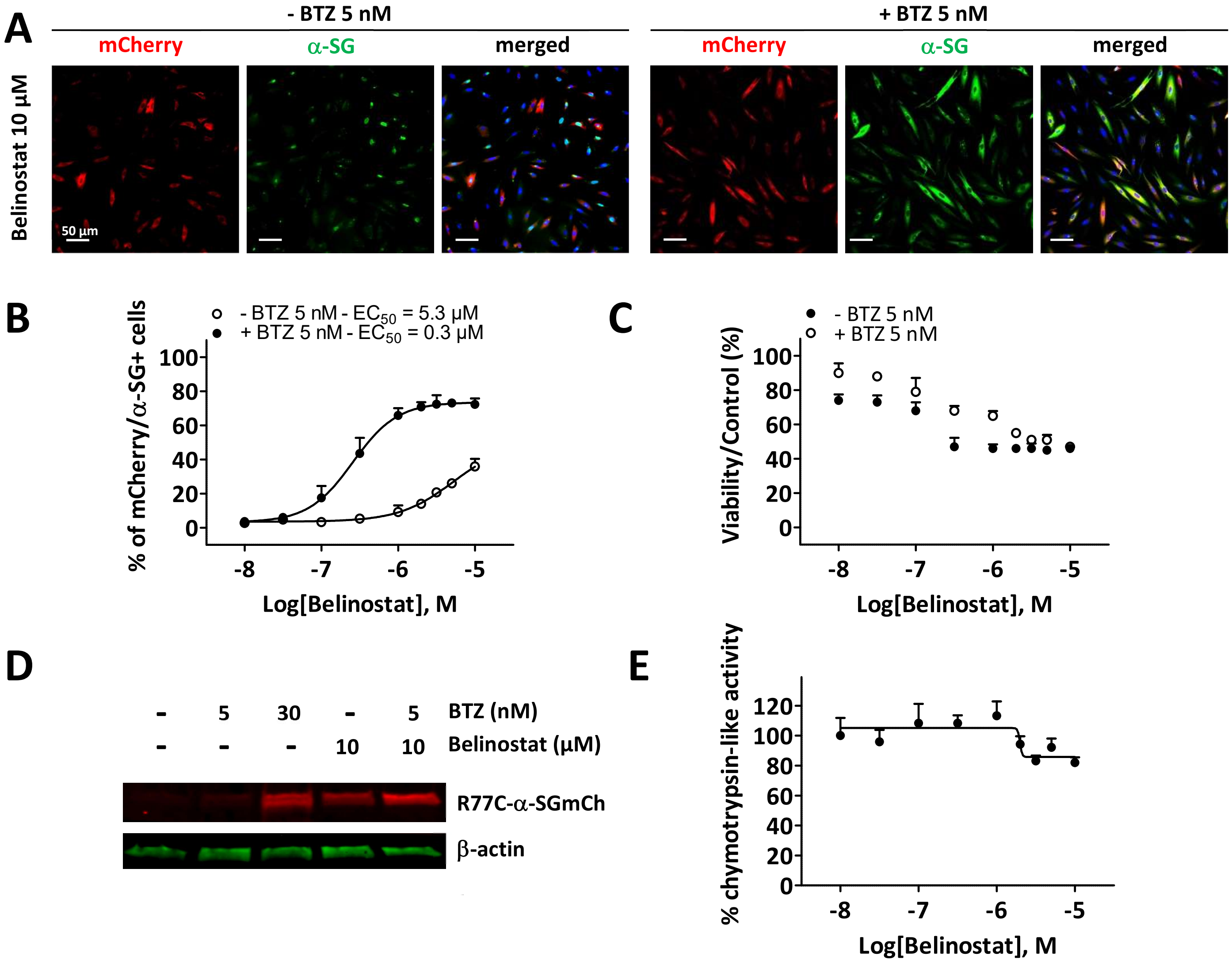

Supplementary Figure S2. Combinatorial effects of bortezomib and belinostat (related to Figure 2). Fibroblasts overexpressing R77C-α-SGmCh were treated with DMSO (0.1%), BTZ (5 nM or 30 nM) or belinostat (10 µM or increasing concentrations) alone or in combination with 5 nM BTZ for 24 hours. (A) mCherry fluorescent signal (red) and α-SG staining (green) after fibroblasts treatments. Nuclei are labelled by Hoechst staining (blue). Scale bar = 50 µm. (B, C) Quantification of mCherry and membrane α-SG positive fibroblasts (B) and cell viability (C) following fibroblasts treatments. Each point represents the mean±SD (n=4) of a representative experiment over 3 independent experiments. (D) Immunoblot analysis of α-SG expression after fibroblasts treatments. β-actin was used to evaluate the loading. (E) Quantification of the chymotrypsin-like activity of proteasome following fibroblasts treatments. Each point represents the mean±SD (n=4) of a representative experiment over 3 independent experiments. BTZ = bortezomib.

Supplementary Figure S3

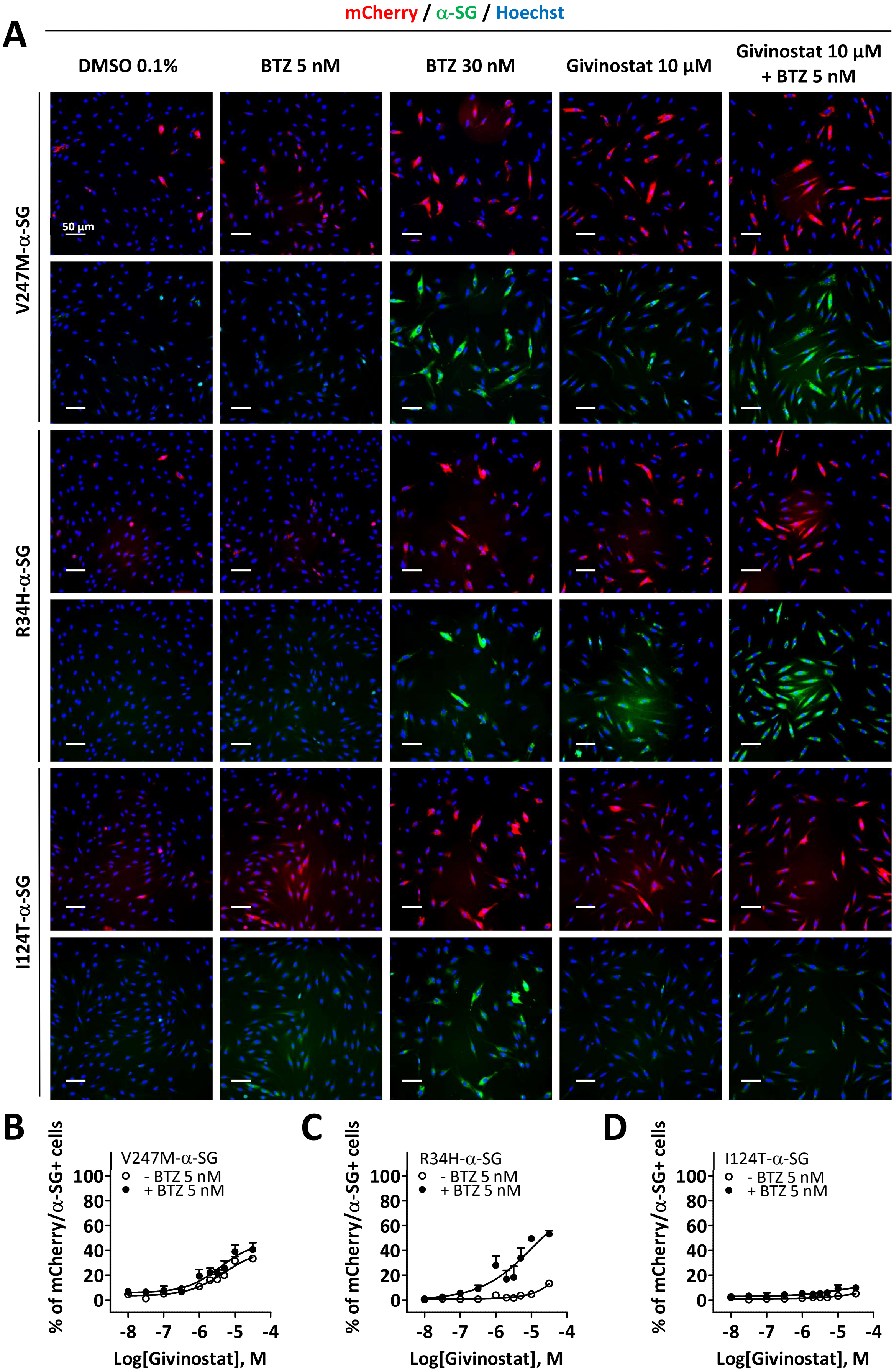

Supplementary Figure S3 Evaluation of givinostat and bortezomib combination on other missense α-SG mutants (related to Figure 3). LGMDR3 patient's fibroblasts were transduced with lentivirus expressing R34H-, I124T- or V247M-α-SGmCh constructs and treated for 24 hours with DMSO (0.1 %), BTZ (5 nM and 30 nM), or increasing concentrations of givinostat alone or in combination with 5 nM BTZ. The membrane α-SG expression was monitored by immunofluorescence under non-permeabilized condition. (A) Images of mCherry fluorescent signal (red), membrane α-SG (green) and Hoechst staining (blue) after fibroblasts treatments. Scale bar = 50 μm. (B-D) Quantification of mCherry and membrane R34H-α-SG (B), I124T-α-SG (C) and V247M-α-SG (D) positive cells following fibroblasts treatments. Each point represents the mean±SD (n=4) of a representative experiment over 3 independent experiments. BTZ = bortezomib.

Supplementary Figure S4

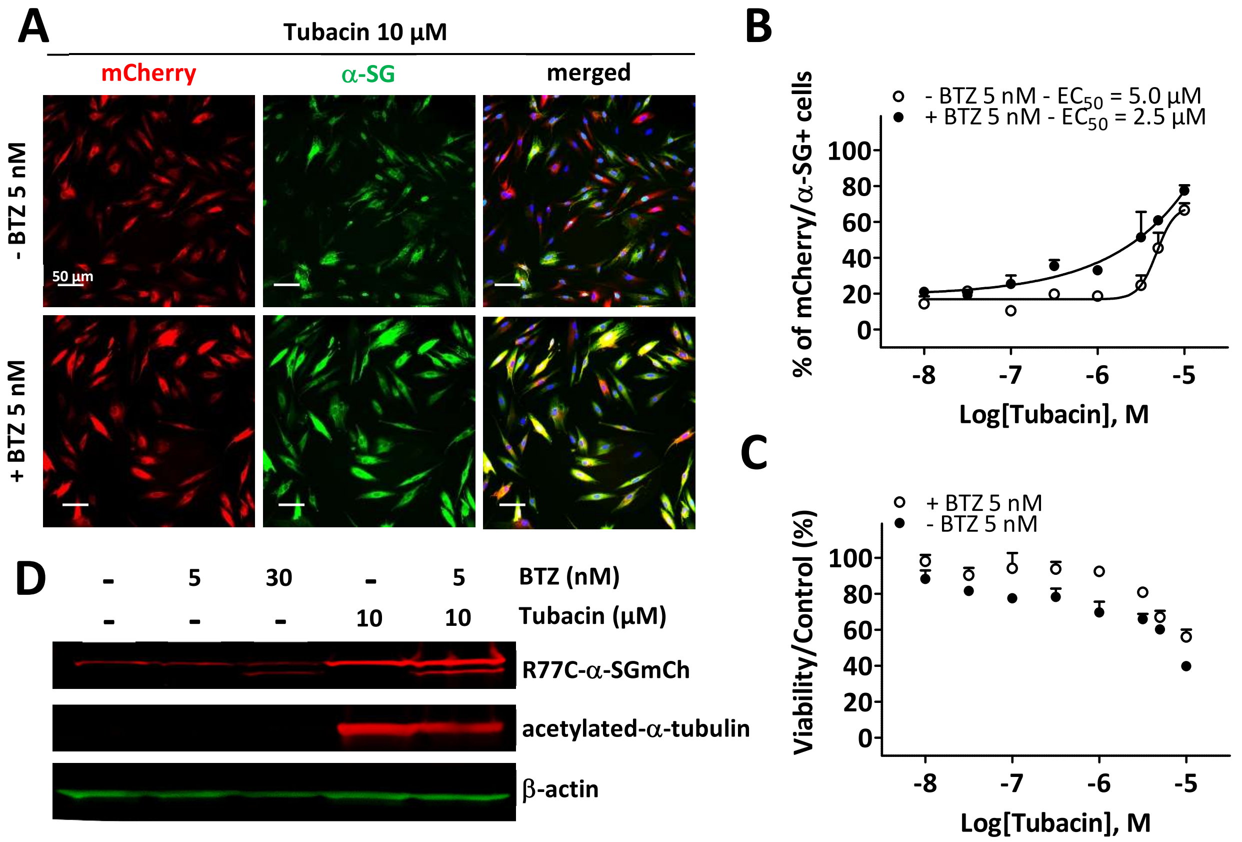

Supplementary Figure S4. Rescue of misfolded R77C-α-SG degradation by tubacin (related to Figure 5). (A-D) Fibroblasts overexpressing R77C-α-SGmCh were treated with DMSO (0.1%), BTZ (5 nM or 30 nM) or tubacin (10 µM or increasing concentrations) alone or in combination with 5 nM BTZ for 24 hours. (A) mCherry fluorescent signal (red) and α-SG staining (green) after fibroblasts treatments. Nuclei are labelled by Hoechst staining (blue). Scale bar = 50 µm. (B, C) Quantification of mCherry and membrane α-SG positive fibroblasts (B) and cell viability (C) following treatments. Each point represents the mean±SD (n=4) of a representative experiment over 3 independent experiments. (D) Immunoblot analysis of α-SG and acetylated-α-tubulin expression in fibroblasts after treatments. β-actin was a loading control.

Supplementary Figure S5

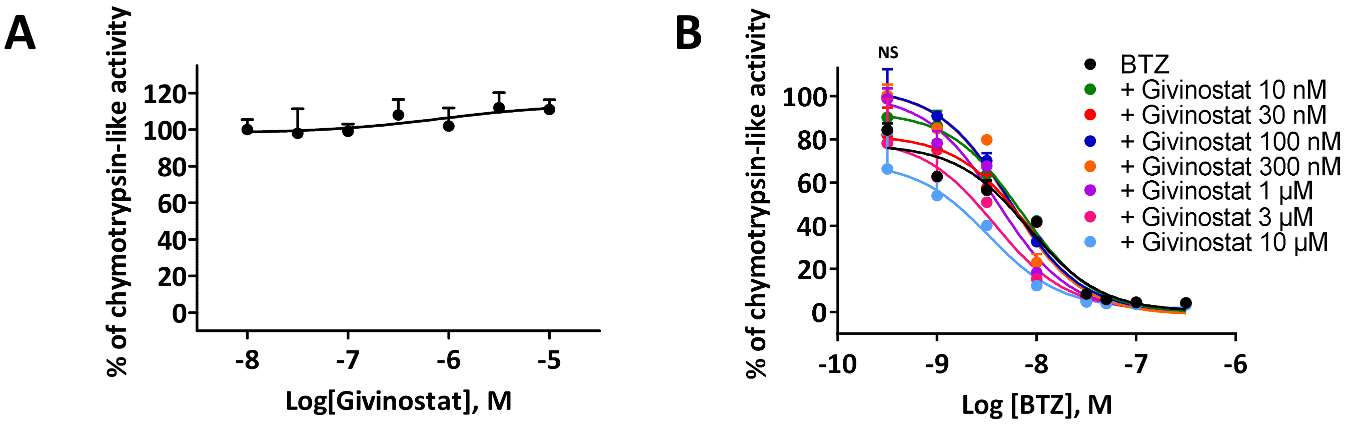

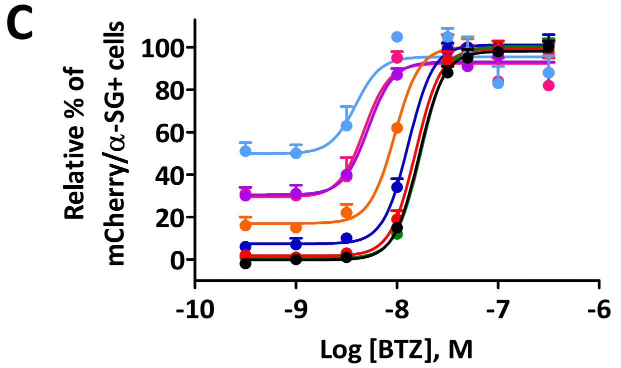

D

Compound	α-SG EC_{50} (nM)	proteasome IC_{50} (nM)
BTZ	16.9	8.3
+ Givinostat 10 nM	16.9	7.1
+ Givinostat 30 nM	14.8	7.9
+ Givinostat 100 nM	12.5	5.3
+ Givinostat 300 nM	9.4	5.3
+ Givinostat 1 µM	5.2	4.1
+ Givinostat 3 µM	3.5	3.7
+ Givinostat 10 µM	3.3	3.3

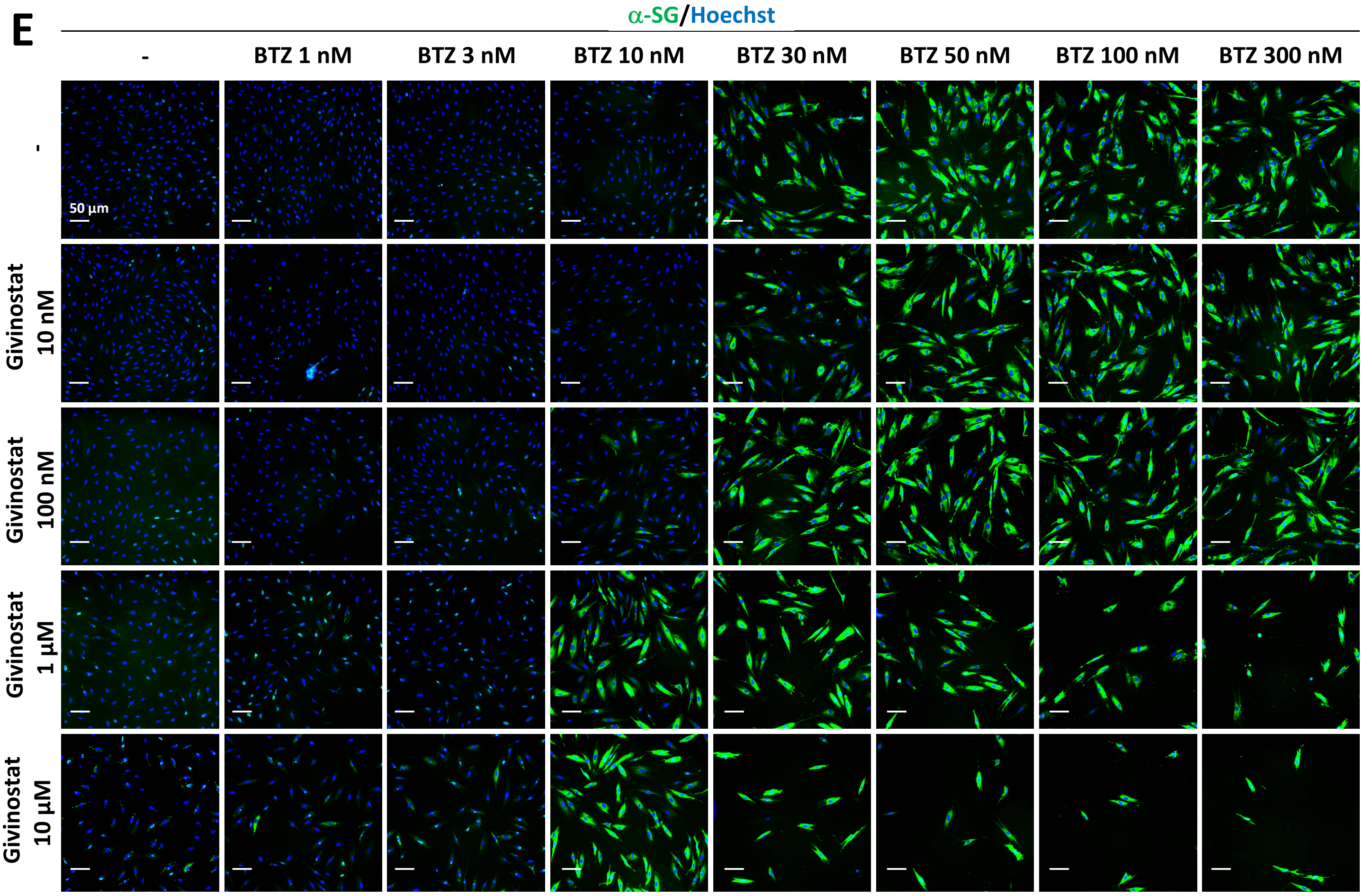

Supplementary Figure S5. Combination of givinostat and bortezomib effects. (A-C) Quantification of chymotrypsin-like activity of proteasome (A-B) and mCherry fluorescent signal and membrane α-SG staining (C) in fibroblasts overexpressing R77C-α-SGmCh and treated with increasing concentrations of givinostat alone or in combination with increasing concentration of BTZ for 24 hours.. Values are expressed as percentage of the response induced by 0.1% DMSO (A-B) or as percentage of the maximal response induced by BTZ (C) and each point represents the mean±SD (n=4) of a representative experiment over 3 independent experiments; NS=non-significant (Student's t-test). (D) EC_{50} and IC_{50} of BTZ and combination treatments on membrane α-SG rescue and proteasome activity. (E) Images of membrane α-SG (green) and Hoechst staining (blue) after treatments. Scale bar = 50 µm; BTZ = bortezomib.

Supplementary Figure S6

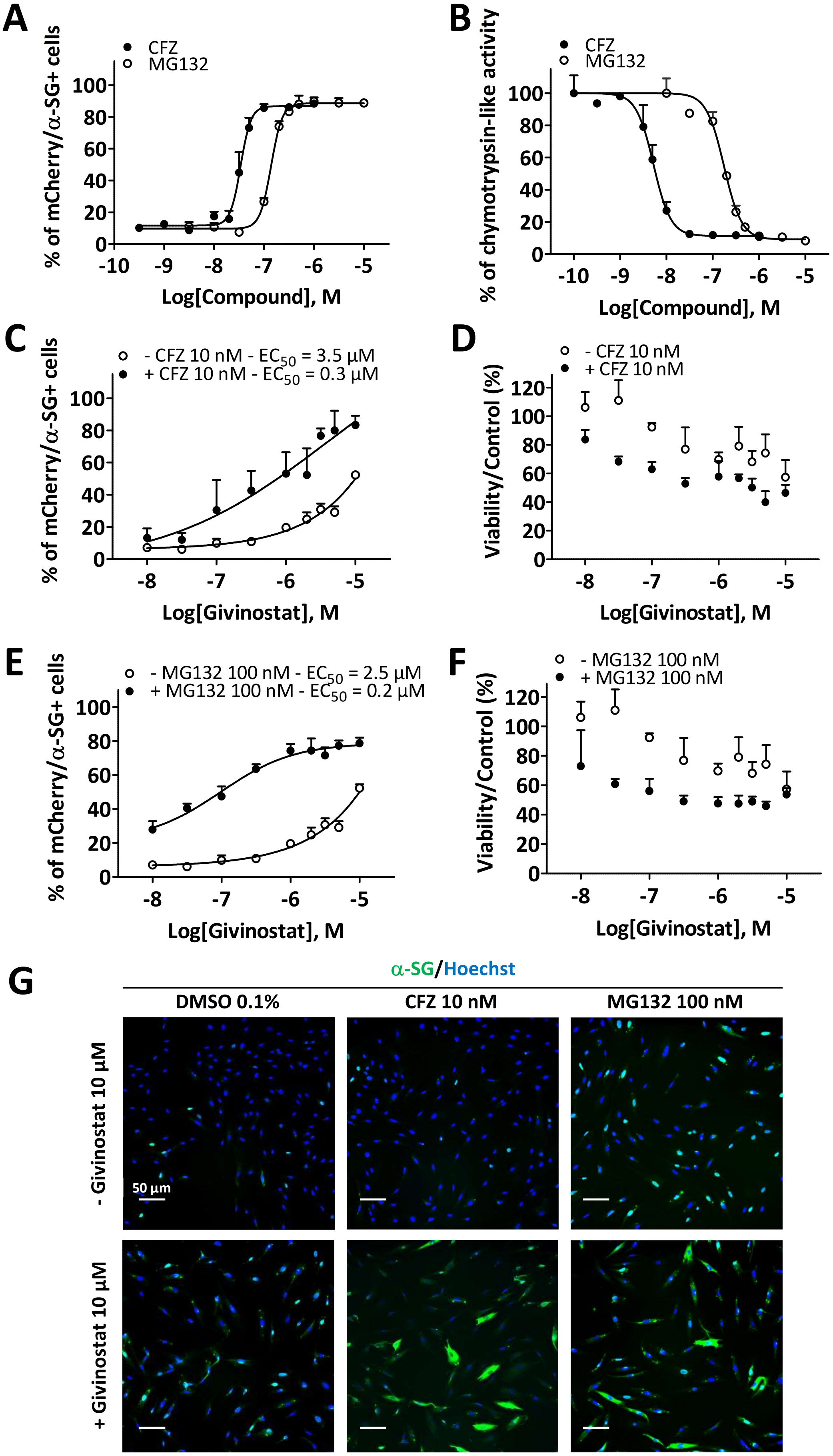

Supplementary Figure S6. Combinatorial effects of givinostat with the proteasome inhibitors carfilzomib and MG132. (A-B) Quantification of mCherry and membrane α-SG positive fibroblasts (A) or chymotrypsin-like activity of proteasome (B) after fibroblasts treatment with increasing concentrations of CFZ or MG132. (C-G) Quantification of mCherry and membrane α-SG positive fibroblasts (C, E), cell viability (D, F) and representative fluorescence images (G) of membrane α-SG (green) and Hoechst staining (blue) after fibroblasts treatments with increasing concentrations of givinostat alone or in combination with 10 nM CFZ (C, D, G) or 100 nM MG132 (E, F, G) for 24 hours. Each point represents the mean±SD (n=4) of a representative experiment over 3 independent experiments. BTZ = bortezomib, CFZ = carfilzomib.

Supplementary Figure S7

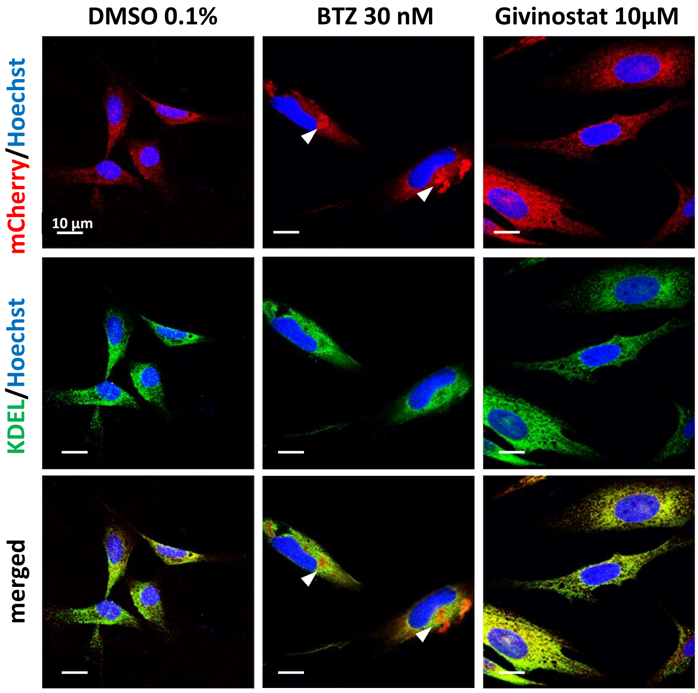

Supplementary Figure S7. R77C-α-SG aggregation in reticulum endoplasmic under proteasome inhibition (related to Figure 6). (A) Confocal images of mCherry fluorescent signal (red), KDEL (green) and Hoechst staining (blue) after DMSO (0.1%), BTZ (30 nM) or givinostat (10 µM) treatments. Scale bar = 10 µm.

Supplementary Table S1

List of the primers used to introduce the R34H, R77C, I124T, V247M, E263K and C283Y mutations (related to Materials and Methods).

Primer name	Primer Sequence
R34H Forward	5'-CACCCACTTGTGGGCCACGTCTTTGTGCACACC-3'
R34H Reverse	5'-GGTGTGCACAAAGACGTGGCCCACAAGTGGGTG-3'
R77C Forward	5'-GCCCCGGTGGCTCTGCTACACCCAGCGC-3'
R77C Reverse	5'-GCGCTGGGTGTAGCAGAGCCACCGGGGC-3'
I124T Forward	5'-CTGGTGCTGGAGACTGGGGACCCAGAA-3'
I124T Reverse	5'-TTCTGGGTCCCCAGTCTCCAGCACCAG-3'
V247M Forward	5'-GTTGACTGGTGCAATATGACCCTGGTGGATA-3'
V247M Reverse	5'-TATCCACCAGGGTCATATTGCACCAGTCAAC-3'
E263K Forward	5'- GCTCACAGAGCCTCTACAAAATCTGTGTGTGTCC-3'
E263K Reverse	5'- GGACACACACAGATTTTGTAGAGGCTCTGTGAGC-3'
C283Y Forward	5'- GGTGTGAGCACCACGTACCAGGAGCACAAC-3'
C283Y Reverse	5'- GGTTGTGCTCCTGGTACGTGGTGCTCACACC-3'

Supplementary Table S2

List of the compounds identified as primary hits during the screening in absence or in presence of 5 nM BTZ and the corresponding percentage of R77C-a-SG rescue. Z-Score and percentage of cell viability. Compounds were considered as primary hits when their Z-Score were superior at 2 standard deviations and their percentage of viability superior at 45% (related to Figure 1).
** values beyond selected thresholds*

	Compound	Family	- BTZ 5 nM			+ BTZ 5 nM		
			% rescue	Z-Score	% viability	% rescue	Z-Score	% viability
Primary hits only in absence of 5 nM BTZ	Istradefylline (KW-6002)	other	93.30	3.43	49.88	67.83	2.50	38.66*
	PHA-665752	other	88.88	3.37	57.14	81.49	2.94	26.19*
	Triptolide	other	78.36	2.94	47.58	52.87	1.92*	58.87
	PF 477736	Kinasei	76.4	2.92	48.63	73.28	2.64	41.10*
	Benazepril hydrochloride	other	73.22	2.81	57.73	40.53	1.43*	70.56
	PF-562271	Kinasei	62.19	2.41	57.2	39.6	1.40*	55.33
	Dasatinib (BMS-354825)	Kinasei	60.90	2.37	93.32	41.47	1.47*	79.08
	VU 0364770	other	57.49	2.26	52.59	25.04	0.83*	53.14
Primary hits only in presence of 5 nM BTZ	JNJ-26481585	HDACi	107.19	4.02	38.84*	95.93	3.47	45.69
	Crizotinib (PF-02341066)	Kinasei	83.83	3.19	44.20*	106.57	3.86	47.89
	AUY922 (NVP-AUY922)	HSP90i	81.42	3.10	38.60*	73.60	2.65	48.81
	ENMD-2076	AKi	80.19	3.06	38.80*	61.17	2.19	50.59
	AZD7762	CDKi	77.76	2.97	40.12*	74.97	2.70	49.88
	BIIB021	HSP90i	74.43	2.85	39.88*	59.39	2.13	45.08
	Ganetespib (STA-9090)	HSP90i	72.40	2.78	39.53*	77.37	2.79	46.46
	PHA-793887	CDKi	68.44	1.95*	54.66	104.94	3.36	48.62
	TG101209	Kinasei	67.13	1.90*	51.86	92.89	2.94	46.74
	ENMD-2076 L-(+)-Tartaric acid	AKi	64.54	1.81*	57.11	70.53	2.15	58.57
	Fedratinib (TG101348)	Kinasei	62.71	1.75*	73.21	98.28	3.13	50.18
	PF-04929113 (SNX-5422)	HSP90i	60.23	1.66*	49.07	79.49	2.46	50.44
	Cyclocytidine HCl	other	50.27	1.99*	49.46	60.63	2.17	45.64
	BX-912	Kinasei	50.01	1.98*	60.58	61.62	2.21	56.57
	Foretinib (GSK1363089. XL880)	other	43.10	1.74*	66.87	74.47	2.68	52.23
	Geldanamycin	HSP90i	35.37	0.78*	49.31	71.33	2.17	49.97
Primary hits in both conditions	CUDC-907	HDACi	103.98	3.20	50.29	105.04	3.37	46.01
	SB939 (Pracinostat)	HDACi	99.38	3.04	59.96	88.65	2.79	56.70
	AZD5438	CDKi	94.93	2.88	48.97	88.81	2.79	56.64
	Dinaciclib (SCH727965)	CDKi	94.08	2.85	56.97	104.81	3.36	56.90
	AT9283	AKi	90.21	3.41	48.87	96.00	3.47	51.41
	ITF2357 (Givinostat)	HDACi	88.24	2.65	59.17	102.93	3.29	49.87
	JTC-801	other	85.63	2.56	59.42	75.51	2.32	50.18
	AR-42 (HDAC-42)	HDACi	85.61	3.18	52.69	76.99	2.86	56.58
	M344	HDACi	80.68	3.07	71.30	78.27	2.82	55.85
	Flavopiridol (Alvocidib) HCl	CDKi	80.43	2.37	52.70	105.72	3.39	46.85
	AT7519	CDKi	78.83	2.32	58.29	96.22	3.05	46.33
	Azilsartan Medoxomil (TAK-491)	other	78.58	2.95	47.23	64.02	2.35	51.94
	TW-37	other	78.35	2.99	66.58	76.65	2.76	60.81
	17-DMAG HCl (Alvespimycin)	HSP90i	77.22	2.95	52.37	79.21	2.85	46.26
	17-AAG (Tanespimycin)	HSP90i	76.60	2.93	53.05	80.15	2.89	51.51
	SNX-2112	HSP90i	73.87	2.14	55.54	77.27	2.38	53.41
	AT13387	HSP90i	73.68	2.83	51.97	57.95	2.07	49.62
	MLN8237 (Alisertib)	AKi	72.86	2.80	57.63	68.17	2.45	47.99
	PCI-24781	HDACi	68.61	2.65	56.59	67.72	2.43	51.56
	Belinostat (PXD101)	HDACi	67.89	2.62	58.22	62.16	2.23	60.09
	JNJ-7706621	CDKi	64.13	2.49	56.84	71.1	2.56	52.38
	Danusertib (PHA-739358)	AKi	59.88	2.33	63.43	75.14	2.71	55.29
	SNS-314 Mesylate	AKi	57.26	2.24	58.95	64.37	2.31	53.20

Supplementary Table S3

Classification of the hit compounds based on a virtual ADMET analysis using artificial intelligence. Final score, ADMET score, activity score and viability score are detailed. (related to Figure 1).

Ranking	Compounds	Family	Final score	ADMET score	Activity score	Viability score
1	PHA-793887	CDKi	0.395	0.773	1.049	0.486
2	Dinaciclib (SCH727965)	CDKi	0.372	0.693	0.941	0.570
3	M344	HDACi	0.368	0.640	0.807	0.713
4	AZD5438	CDKi	0.329	0.707	0.949	0.490
5	AT7519	CDKi	0.325	0.533	0.788	0.583
6	SB939 (Pracinostat)	HDACi	0.318	0.587	0.994	0.600
7	CUDC-907	HDACi	0.307	0.573	1.040	0.503
8	ITF2357 (Givinostat)	HDACi	0.299	0.640	0.882	0.592
9	Istradefylline (KW-6002)	other	0.298	0.547	0.933	0.499
10	TW-37	other	0.285	0.640	0.784	0.666
11	AT9283	Aki	0.282	0.760	0.902	0.489
12	JNJ-7706621	Aki	0.277	0.667	0.641	0.568
13	ENMD-2076 L-(+)-Tartaric acid	Aki	0.275	0.587	0.705	0.586
14	AR-42 (HDAC-42)	HDACi	0.265	0.600	0.856	0.527
15	JNJ-26481585	HDACi	0.263	0.507	0.959	0.457
16	Crizotinib (PF-02341066)	Kinasei	0.259	0.440	1.066	0.479
17	Dasatinib (BMS-354825)	Kinasei	0.250	0.507	0.609	0.933
18	Fedratinib (TG101348)	Kinasei	0.250	0.587	0.983	0.502
19	Benazepril hydrochloride	Other	0.248	0.640	0.732	0.577
20	AT13387	HSP90i	0.245	0.613	0.737	0.520
21	Belinostat (PXD101)	HDACi	0.242	0.600	0.679	0.582
22	PF-04929113 (SNX-5422)	HSP90i	0.241	0.560	0.795	0.504
23	MLN8237 (Alisertib)	Aki	0.235	0.653	0.729	0.576
24	Ganetespib (STA-9090)	HSP90i	0.235	0.573	0.774	0.465
25	17-AAG (Tanespimycin)	HSP90i	0.233	0.613	0.766	0.531
26	Danusertib (PHA-739358)	Aki	0.233	0.573	0.599	0.634
27	17-DMAG HCl (Alvespimycin)	HSP90i	0.232	0.533	0.772	0.524
28	TG101209	Kinasei	0.232	0.453	0.929	0.467
29	JTC-801	other	0.231	0.453	0.856	0.594
30	PHA-665752	other	0230	0.613	0.889	0.571
31	AZD7762	CDKi	0.229	0.587	0.750	0.499
32	PCI-24781	HDACi	0.228	0.533	0.686	0.566
33	Flavopiridol (Alvocidib) HCl	CDKi	0.226	0.813	0.804	0.527
34	Cyclocytidine HCl	other	0.225	0.547	0.606	0.456
35	SNX-2112	HSP90i	0.224	0.587	0.739	0.555
36	Triptolide	other	0.219	0.587	0.784	0.476
37	PF 477736	other	0.218	0.587	0.764	0.486
38	Azilsartan Medoxomil (TAK-491)	other	0.218	0.587	0.786	0.472
39	Geldanamycin	HSP90i	0.209	0.587	0.713	0.500
40	AUY922 (NVP-AUY922)	HSP90i	0.201	0.560	0.736	0.488
41	PF-562271	Kinasei	0.190	0.533	0.622	0.572
42	VU 0364770	other	0.185	0.613	0.575	0.526
43	SNS-314 Mesylate	Aki	0.185	0.547	0.573	0.590
44	BIIB021	HSP90i	0.182	0.680	0.594	0.451
45	ENMD-2076	AKi	0.177	0.573	0.612	0.506
46	BX-912	Kinasei	0.177	0.507	0.616	0.566
47	Foretinib (GSK1363089. XL880)	other	0.171	0.440	0.745	0.522
